# Translating evidence into practice: recommendations by a UK expert panel on the use of aflibercept in diabetic macular oedema

**DOI:** 10.1038/s41433-019-0615-8

**Published:** 2019-10-16

**Authors:** Ian Pearce, Clare Bailey, Emily Fletcher, Faruque Ghanchi, Christina Rennie, Cynthia Santiago, Jackie Napier, Yit Yang

**Affiliations:** 1 Royal Liverpool University Hospital, Liverpool, UK; 2 University Hospitals Bristol NHS Foundation Trust, Bristol, UK; 3 Gloucestershire Hospitals NHS Foundation Trust, Gloucester, UK; 4 Bradford Teaching Hospitals NHS Foundation Trust, Bradford, UK; 5 Southampton General Hospital, Southampton, UK; 6 Aberdeen Royal Hospitals NHS Trust, Aberdeen, UK; 7 Medical Affairs, Bayer Plc, Reading, UK; 8 The Royal Wolverhampton NHS Trust, Wolverhampton, UK

**Keywords:** Drug therapy, Therapeutics

## Abstract

**Objectives:**

This paper describes recommendations from a panel of UK retina experts on aflibercept in diabetic macular oedema (DMO).

**Methods:**

A roundtable meeting was held in London, UK in March 2018. The meeting was sponsored by Bayer.

**Results:**

Recommendations are based on clinical experience and level 1 evidence. Clinical experience supports the evidence base, reinforcing that aflibercept should be initiated with intensive proactive dosing at 2 mg every 4 weeks. Most panel members use six initial 4-weekly doses as in Protocol T, rather than five initial monthly doses as recommended in the Summary of product characteristics (SmPC). After intensive proactive dosing, patients with a good response (meet Protocol T ‘improvement’ criteria ≥5-letter improvement in visual acuity [VA] and/or ≥10% improvement in central subfield thickness [CST] from baseline) but who are not yet stable should continue with 4-weekly aflibercept until stability is reached. Patients with a good response and stability should initiate monitor-and-extend (not in line with SmPC). Those with a sub-optimal response (meet ‘improvement’ criteria but with additional concerns e.g. fluid worsening on macular volume map) should continue with 4-weekly aflibercept but additional treatments should be considered (aflibercept is not licensed for combination treatment). For patients with no response (no change, or meeting Protocol T ‘worsening’ criteria [≥5-letter decrease in VA and/or ≥ 10% increase in CST] from baseline), switching to a non-anti-vascular endothelial growth factor treatment should be considered.

**Conclusions:**

Clinical experience reinforces that, when using aflibercept in DMO, the licensed posology or Protocol T regimens achieve the best outcomes.

## Introduction

Diabetic macular oedema (DMO) is characterised by accumulation of fluid from leaking blood vessels [1], caused by breakdown of the blood-retinal barrier [2]. DMO is often associated with exudates and can lead to blurring and distortion of central vision [2]. It represents the most common cause of visual impairment in patients with diabetes, accounting for around 75% of cases of vision loss [3].

Aflibercept is a recombinant fusion protein, comprising portions of human vascular endothelial growth factor receptor (VEGFR)-1 and VEGFR-2 extracellular domains that are fused to the Fc portion of human immunoglobulin G1 [4]. Aflibercept is approved for the treatment of visual impairment due to DMO; the licensed posology is shown in Table 1 and Appendix Fig. 1 [4]. Based on the results from the Protocol T trial [5, 6], guidelines from the European Society of Retina Specialists (EURETINA), published in 2017, state that ‘*aflibercept is the drug of choice in DMO eyes with baseline best-corrected visual acuity (BCVA) below 69 letters, as it shows superiority to bevacizumab over 2 years and ranibizumab in the first year of treatment*’. Based on the evidence available to date, the guidelines suggest that it is currently ‘an open choice’ as to whether aflibercept loading doses should be followed by fixed bimonthly injections or a pro re nata (PRN) regimen with monthly (4-weekly) monitoring [2]. It should be noted that the treatment regimen used for aflibercept in Protocol T is not in accordance with the UK Summary of Product Characteristics (SmPC). Furthermore, the 0.3 mg dose of ranibizumab is not licensed in the UK, and bevacizumab is not licensed for the treatment of retinal disease.

**Table 1:** European posology of aflibercept in diabetic macular oedema [4]

The recommended dose for Eylea is 2 mg aflibercept equivalent to 50 ml.
Eylea treatment is initiated with one injection per month for five consecutive doses, followed by one injection every two months. There is no requirement for monitoring between injections.
After the first 12 months of treatment with Eylea, and based on visual and/or anatomic outcomes, the treatment interval may be extended, such as with a treat-and-extend dosing regimen, where the treatment intervals are gradually increased to maintain stable visual and/or anatomic outcomes; however there are insufficient data to conclude on the length of these intervals. If visual and/or anatomic outcomes deteriorate, the treatment interval should be shortened accordingly.
The schedule for monitoring should therefore be determined by the treating physician and may be more frequent than the schedule of injections.
If visual and anatomic outcomes indicate that the patient is not benefiting from continued treatment, Eylea should be discontinued.

To date, the protocols used in clinical trials in DMO have varied between trials. Translating clinical trial regimens and the aflibercept licensed posology into clinical practice is therefore difficult and has led to some confusion. Gathering expert opinion on existing data via a roundtable consensus is a widely accepted means of translating clinical trials into practice. This paper therefore describes recommendations from a roundtable of retina experts on the use of aflibercept for management of visual impairment due to DMO, based on current evidence and practical experience in the UK. For effective implementation of these recommendations, several considerations should be borne in mind. First, it is anticipated that the recommendations will apply to the majority of patients. Those who fall outside these recommendations (e.g. those who have another comorbidity in the eye) should be treated on a case-by-case basis. Second, the recommendations provide the minimum standard, based on a consensus of retina experts. They should be tailored to each unit, taking local protocols into consideration. Third, given that most clinics are organised using weekly rather than monthly schedules, ‘4-weekly’ and ‘monthly’ (and likewise ‘8-weekly’ and ‘bimonthly’) are considered interchangeable for the purposes of the recommendations. Fourth, throughout DMO treatment, the underlying systemic disease should be managed effectively by the appropriate healthcare professional. Finally, the discussion is limited to aflibercept only. Other agents approved for DMO were not discussed during the roundtable meeting and are not covered in the paper.

## Aflibercept in DMO: funding criteria in the UK

These recommendations apply to patients with centre-involving DMO who are eligible for National Health Service (NHS)-funded aflibercept treatment.

In England and Wales, aflibercept is recommended by the National Institute for Health and Care Excellence (NICE; Technology Appraisal 346) as an option for treating visual impairment caused by DMO only if the eye has a central retinal thickness of 400 µm or more at the start of treatment and the company provides aflibercept with the discount agreed in the patient access scheme [7]. The Northern Ireland Department of Health has fully endorsed the NICE Technology Appraisal for aflibercept in DMO [8].

In Scotland, aflibercept is accepted for use by the Scottish Medicines’ Consortium (SMC) for the treatment of visual impairment due to DMO in adults with a BCVA of 75 Early Treatment Diabetic Retinopathy Study letters or less at baseline. Use of aflibercept is contingent upon the continuing availability of the patient access scheme, or a list price that is equivalent or lower, in NHS Scotland [9].

## Roundtable meeting

The structured roundtable meeting was held in London, UK on March 7, 2018. The meeting was sponsored by Bayer and attended by a panel of seven retina specialists with experience in treating DMO with aflibercept. Prior to the meeting, the panel members were asked to provide information on their clinic size, clinic setup and aflibercept treatment protocols. This information was presented at the meeting and was used to initiate and guide the discussion.

## Patients considered unsuitable for anti-VEGF treatment

While these recommendations are specific to patients eligible for aflibercept, the authors felt it would be of value to identify patients who may not be suitable for anti-VEGF treatment. These include those who are pregnant, are trying to get pregnant, have refused anti-VEGF treatment, or have contraindications e.g. an allergic reaction to anti-VEGFs.

## Data on aflibercept in DMO

Table 2 provides a summary of key data with aflibercept in DMO, including: the pivotal phase III VIVID and VISTA trials (comparing the efficacy of aflibercept and laser) [10–12]; Protocol T (a head-to-head trial comparing aflibercept, ranibizumab and bevacizumab [5]); and two real-world single-centre studies reporting on the use of aflibercept administered according to licence [13, 14].

**Table 2:** Summary of key data with aflibercept in DMO

Trial	Patients	Study design	Intervention	Primary endpoint	Key findings
VIVID/VISTA [10–12]	Patients with centre-involving DME and BCVA between 73 and 24 letters [10]	Phase III, randomised, double-blind, multicentre trials conducted in the USA (VISTA) or Europe, Japan and Australia (VIVID) [10]	• From baseline to week 52, randomised to one of three treatment arms [10]; ◦ Aflibercept 2q4 ◦ Aflibercept 2q8 after five initial monthly doses ◦ Laser • After week 100, patients in the laser group were eligible to receive aflibercept PRN^a^ [12]	Mean change in BCVA from baseline to week 52 [10]	• Both aflibercept 2q4 and 2q8 yielded significantly greater gains in BCVA (10.7 and 10.5 letters, respectively, in VIVID; 12.5 and 10.7 letters, respectively, in VISTA) than laser (1.2 letters in VIVID; 0.2 letters in VISTA; *p* < 0.0001 for all aflibercept groups vs. laser) at week 52 [10] • BCVA gains were maintained through week 100 [11] and week 148 [12] • Reductions in CRT were significantly greater with aflibercept than with laser at week 52 [10], and were maintained through weeks 100 [11] and 148 [12]
Protocol T [5, 6]	Patients with centre-involving DME and BCVA between 78 and 24 letters [5]	Randomised head-to-head trial conducted in the USA [5]	• One of three treatment arms in a PRN regimen with strict retreatment criteria: [5] ◦ Aflibercept (2 mg)^b^ ◦ Ranibizumab (0.3 mg)^c^ ◦ Bevacizumab (1.25 mg)^d^ • Patients were treated intensively up to week 20; treatment administered every 4 weeks unless VA 20/20 and CST < 250 µm, and no improvement^e^ or worsening^f^ in response to the last two injections (i.e. stability established) [5] • From week 24, If stable, the treatment interval could be extended; irrespective of whether VA 20/20 or CST < 250 µm had been achieved^g^ [5] • Monitoring conducted every 4 weeks during Year 1 [5] • In Year 2, an M&E strategy was employed^h^ [6]	Change in VA from baseline to week 52	***Year 1*** [5] • Mean gain in VA was significantly higher with aflibercept (13.3 letters) than with ranibizumab (11.2 letters; *p* = 0.03) or bevacizumab (9.7 letters; *p* < 0.001) • In patients with baseline VA worse than 20/40, aflibercept was associated with significantly greater VA gains than ranibizumab (*p* = 0.001) or bevacizumab (*p* = 0.003) • VA gains did not differ significantly between groups in patients with a baseline VA of 20/32 to 20/40 • In the overall population, the median number of injections in Year 1 was lower with aflibercept than with ranibizumab or bevacizumab (9, 10 and 10, respectively) ***Year 2*** [6] • VA gains were maintained to Year 2: mean VA gain from baseline to 2 years of 12.8 letters for aflibercept, 12.3 letters for ranibizumab, and 10.0 letters for bevacizumab (aflibercept vs. ranibizumab: *p* = 0.47; aflibercept vs. bevacizumab: *p* = 0.02) • Significantly greater VA gains were achieved with aflibercept than bevacizumab (*p* = 0.02) in patients with baseline VA worse than 20/40 • Median number of injections in Year 2 was lower for aflibercept than for ranibizumab or bevacizumab (5, 6 and 6, respectively) • Total median number of injections over 2 years was 15, 16 and 16, respectively (2-year completers only)
Campos Polo et al. [13]	Anti-VEGF-naïve patients with clinically significant DMO (*n* = 15)	Real-world single-centre study	Aflibercept 2 mg for five monthly doses followed by dosing every 2 months from baseline to month 12	Mean change in BCVA from baseline to week 52	• From baseline to 12 months, mean BCVA increased by 14.9 letters and central macular thickness reduced by 231.5 µm (*p* < 0.001 for both analyses)
Moorfields Eye Hospital audit [14]	Patients with DMO (*n* = 79^i^)	Real-world single-centre study: Audit of DMO data from Moorfields Eye Hospital in London	Aflibercept 2 mg for five monthly doses followed by dosing every 2 months	Not reported	• Mean BCVA increased by 7.8 letters from baseline to 6 months (*p* = 0.01), with a mean of 4.8 injections • Mean BCVA increased by 8.3 letters from baseline to 12 months (p-value not reported), with a mean of 7.0 injections • Mean CRT and mean macular volume decreased from baseline to 6 months, and were maintained to 12 months (values not reported)

*2q4* 2 mg every 4 weeks, *2q8* 2 mg every 8 weeks, *BCVA* best-corrected visual acuity, *CRT* central retinal thickness *CST* central subfield thickness, *DMO* diabetic macular oedema, *M&E* monitor-and-extend, *OCT* optical coherence tomography, *PRN* pro re nata, *VA* visual acuity, *VEGF* vascular endothelial growth factor

^a^Retreatment criteria were defined as: (a) a > 50 mm increase in CST compared with the lowest previous measurement; (b) new or persistent cystic retinal changes or subretinal fluid on OCT, or persistent diffuse oedema in the central subfield on OCT; (c) a loss of ≥5 letters in BCVA from the best previous measurement in conjunction with any increase in CST; or (d) an increase of ≥5 letters in BCVA between the current and the most recent visit

^b^The Protocol T aflibercept regimen differs from the regimen in the UK Summary of Product Characteristics

^c^The 0.3 mg dose of ranibizumab is not licensed in the UK

^d^Bevacizumab is not licensed for the treatment of retinal disease

^e^Improvement was defined as ≥ 5-letter improvement in VA or ≥10% improvement in CST from baseline

^f^Worsening was defined as ≥5-letter decrease in VA or ≥10% increase in CST from baseline

^g^Injections were withheld if there was no improvement or worsening after two injections (i.e. stability was maintained). Injections were reinitiated if VA or CST worsened. If treatment was reinitiated, injections continued every 4 weeks until there was no improvement or worsening after two injections (i.e. stability was re-established). Focal/grid laser was initiated in eyes with (a) CST ≥ 250 μm or oedema that was threatening the fovea and (b) no improvement in VA or CST over the last two injections.

^h^M&E involved doubling the extension of the monitoring visits if the stability criteria were met (i.e. every 4 weeks extended to every 8 weeks then every 16 weeks, if applicable, up to a maximum of 16 weeks)

^i^Number of patients completing 12 months of follow-up

## Aflibercept regimens

There are three aflibercept regimens that can be followed in DMO: the licensed posology (mentioned above); Protocol T (PRN with 4-weekly monitoring followed by monitor-and-extend [M&E]; outlined in Table 2); treat-and-extend (T&E; outlined below, currently under investigation in an ongoing trial). An overview of each regimen is shown below.

### Licensed posology: proactive dosing in Year 1 with the option of extension in Year 2

The European posology for aflibercept in DMO is shown in Table 1 and Appendix Fig. 1 [4]. Treatment should begin with intensive proactive dosing—one 2 mg injection every month for five consecutive doses—followed by one injection every 2 months. After the first 12 months, the treatment interval may be extended. The monitoring schedule should be determined by the treating physician.

### Protocol T: PRN with 4-weekly monitoring in Year 1 followed by M&E in Year 2

The Protocol T aflibercept regimen is described in Table 2, and comprises PRN with 4-weekly monitoring in Year 1 (with extension of intervals once stability has been reached) and M&E in Year 2 [5, 6] (not in line with licensed posology [4]).

The aflibercept PRN regimen in Protocol T allowed for extension of monitoring intervals in Year 2 [6]. One may, however, choose to continue with ongoing 4-weekly monitoring, as described in the ranibizumab RESTORE trial [15] (which formed the basis of UK DMO management previously). In clinical practice, however, this option is unlikely to be practical.

### T&E after initial loading: proactive dosing with extended treatment intervals

The aflibercept SmPC states that, after 12 months of DMO treatment, a proactive T&E regimen may be initiated (note that initiating aflibercept T&E immediately after loading is not in line with licensed posology [4]). During T&E, the treatment interval can be extended (or reduced, if required), based on visual and anatomic criteria. The monitoring schedule should be determined by the treating physician [4].

There is currently limited evidence available on the use of aflibercept T&E in DMO. At present, the recommendation to consider T&E as an option in DMO is based on level 5 evidence (expert opinion) only. An ongoing phase IV trial (VIOLET) aims to compare the efficacy of this regimen (and PRN) versus fixed dosing every 2 months (NCT02818998). The study is due to be completed in 2019.

## Monitoring considerations

As well as VA and CST, changes in the macular volume map should be considered when making aflibercept treatment decisions in DMO (unlike in neovascular AMD). Changes in the macular volume map, and trends towards increasing macular volume, may precipitate a change in treatment.

Recommendations for incorporating the macular volume map into treatment decisions are given below.

## Expert panel recommendations

The recommendations below are derived from clinical experience based on level 1 evidence.

### Starting aflibercept: begin with intensive proactive dosing

**Fig. 1:**
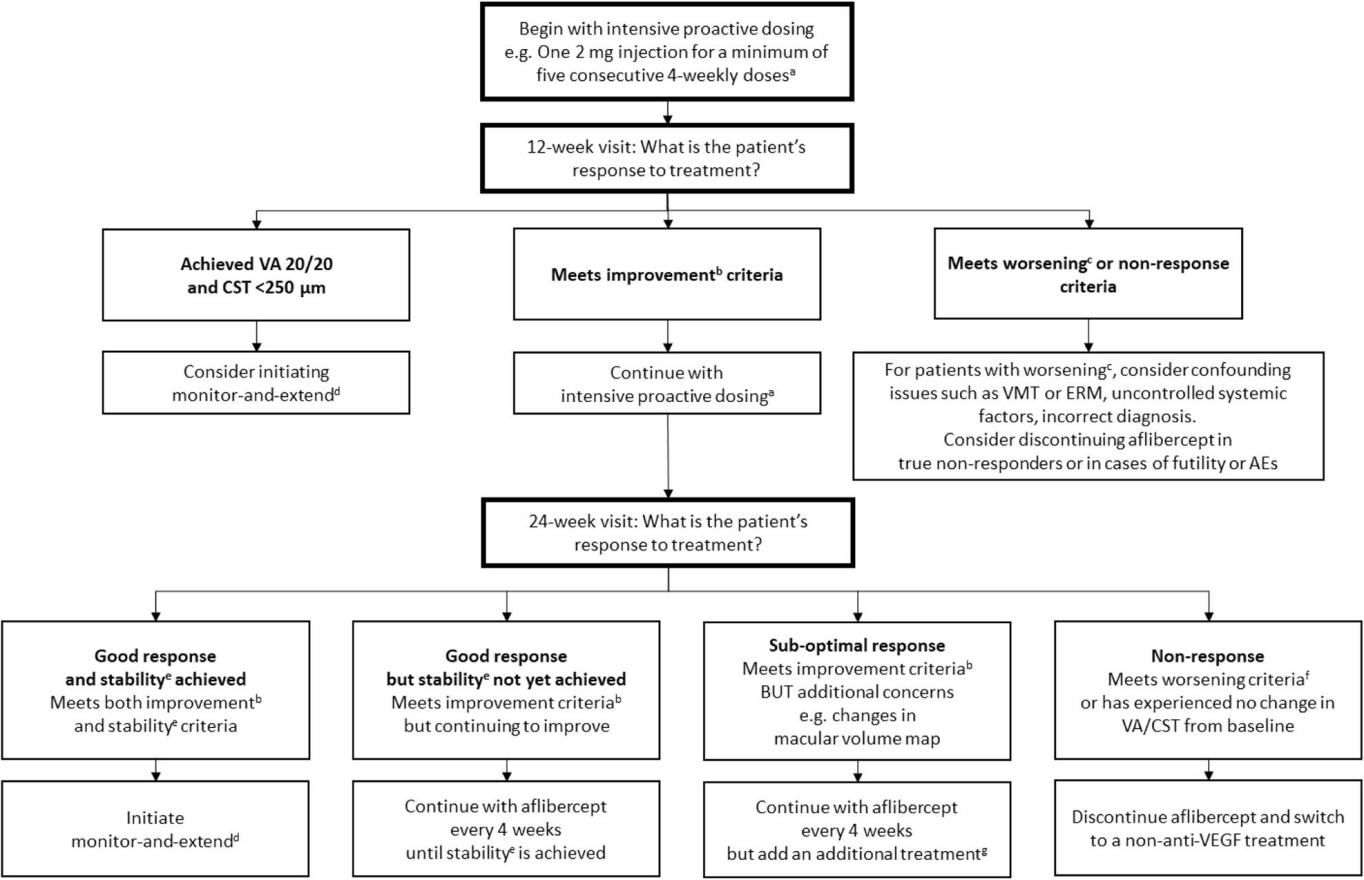
Algorithm for the treatment of diabetic macula oedema with aflibercept. ^a^The aflibercept SmPC states that treatment should be initiated with one injection per month for five consecutive doses, followed by one injection every two months. Greater than 5 monthly injections in Year 1 is not in line with the licensed posology [4]; ^b^Improvement defined as in Protocol T: ≥5-letter improvement in VA and/or ≥10% improvement in CST from baseline [5]; ^c^≥5-letter decrease in VA from baseline; ^d^Initiating M&E during or immediately after loading is not in line with the aflibercept SmPC, which recommends bimonthly dosing until 12 months [4]; ^e^Stability defined as in Protocol T: no improvement or worsening in both VA and CST after two consecutive injections [5]; ^f^Worsening defined as in Protocol T: ≥5-letter decrease in VA and/or ≥10% increase in CST from baseline [5]; ^g^Aflibercept is not licensed for use in combination with any other drug treatments or laser [4]. Optical coherence tomography and fundus fluorescein angiography may help to guide the decision of which additional treatment is most appropriate to use e.g. laser, steroids, etc. AE adverse event, CST central subfield thickness, ERM epiretinal membranes, M&E monitor-and-extend, SmPC Summary of product characteristics, VA visual acuity, VMT vitreomacular traction

Clinical experience supports the evidence base and reinforces that aflibercept treatment should be initiated with intensive proactive dosing at 2 mg every 4 weeks (Fig. 1). The majority of attendees use six initial 4-weekly doses in line with Protocol T [5], rather than the five initial monthly doses as recommended in the SmPC [4]. Most patients will require intensive proactive dosing at the start of treatment to achieve their ‘best’ response.

The importance of an intensive loading phase is supported by a post hoc analysis of pooled VIVID and VISTA data (*n* = 576), in which the proportion of eyes gaining ≥5 letters increased with each subsequent aflibercept injection. From injection 1 to 2, 60% of eyes gained ≥5 letters, 19% of the remaining eyes gained ≥5 letters from injection 2 to 3, 16% from injection 3 to 4, and 15% from injection 4 to 5 [16].

During aflibercept treatment, the panel members recommend monitoring at a minimum at baseline, 12 weeks, 24 weeks, and whenever a treatment decision is due to be made. More frequent monitoring may be required in patients with severe diabetic retinopathy, bilateral disease or other risk factors.

During the aflibercept loading phase, ‘improvement’, ‘worsening’ and ‘stability’ are defined as in Protocol T i.e. improvement: ≥5-letter improvement in VA and/or ≥10% improvement in CST; worsening: ≥5-letter decrease in VA and/or ≥10% increase in CST; stability: no improvement or worsening in both VA and CST after two consecutive injections [5].

The purpose of the 12-week visit is to assess early response to aflibercept. At this point, one may consider initiating aflibercept M&E in patients who have already achieved both VA 20/20 and CST <250 µm. In Protocol T, only 7/208 patients (3.4%) achieved such ‘treatment success’ before the end of the loading phase (i.e. required fewer than the mandated six loading injections) [5]. It should be noted that this approach is not in line with the aflibercept SmPC, which recommends five proactive loading doses, irrespective of whether such goals have been achieved [4]. Patients who are improving at the 12-week visit should continue with intensive proactive dosing with aflibercept. In patients with worsening of VA and/or CST, further investigations should be conducted to confirm that the fluid is due to diabetic maculopathy. If so, the patient should be considered a non-responder and aflibercept treatment should be discontinued. In reality, very few patients would be considered true non-responders at this point; most would, at worst, be deemed sub-optimal responders in whom continued aflibercept treatment would be beneficial. Aflibercept treatment discontinuation at this point should also be considered in cases of futility (e.g. structural damage that would mean treatment would not improve vision) or in patients with an adverse event (AE).

At the 24-week visit, response to aflibercept treatment should be assessed in order to determine continued management (Fig. 1). One should ensure that adequate and timely loading has been completed before defining a patient’s response to aflibercept. Patients with a good response but who are not yet stable should continue with 4-weekly aflibercept until stability is reached. Patients with a good response and stability should initiate aflibercept M&E (not in line with SmPC). Those with a sub-optimal response should continue with 4-weekly aflibercept but additional treatments should be considered (aflibercept is not licensed for combination treatment). For patients with no response to aflibercept, switching to a non-anti-VEGF treatment should be considered. These strategies, and the respective definitions of response, are described in more detail below.

The definition of response should take into consideration changes in, and stability of, VA and fluid over the entire treatment course (e.g. during the entire loading phase, as opposed to looking at the previous visit only). When considering changes in VA, the managing ophthalmologist should confirm that the change is indeed attributable to improvement or worsening of DMO and not due to another factor e.g. cataract removal.

### Good response to aflibercept treatment but VA and/or CST continuing to improve: continue with aflibercept until stability is achieved

Patients who meet the ‘improvement’ criteria but who have not yet reached stability should continue to receive aflibercept every 4 weeks, until stability is achieved (Fig. 1). Continuing with 4-weekly aflibercept dosing after the fifth loading dose is not in line with SmPC posology, which recommends one injection per month for five consecutive doses, followed by one injection every two months [4].

### Good response to aflibercept treatment and stability achieved: initiate an aflibercept M&E strategy

At any period after the intensive proactive loading phase, an aflibercept M&E strategy (similar to Protocol T Year 2 [6], described above under ‘*Protocol T: PRN with 4-weekly monitoring in Year 1 followed by M&E in Year 2*’) should be considered for patients with a good response to treatment and stable disease (Fig. 1). It should be noted that initiating aflibercept M&E immediately after loading is not in line with SmPC posology, which recommends bimonthly dosing until 12 months, with the option of extending the treatment intervals thereafter [4]. At the beginning of M&E, the VA and CST values of interest are reset; during the loading phase, treatment decisions should be based on improvement/worsening *versus baseline*. During M&E, however, treatment decisions should be based on the VA and CST at the point at which the patient was considered stable i.e. when the decision was made to defer injections.

The support for an aflibercept M&E strategy comes from Protocol T; here, the number of aflibercept injections required to maintain good visual outcomes decreased from Year 1 (nine injections) [5] to 2 (five injections) [6], indicating that some/many/all patients with disease control can transition to less treatment/monitoring as they pass along the management pathway.

During aflibercept M&E, the time between visits should be extended by doubling the monitoring periods based on visual and anatomic criteria (i.e. every 4 weeks doubled to every 8 weeks if stable, and then every 16 weeks if still stable, up to a maximum of 16 weeks), as in Protocol I (ranibizumab or triamcinolone plus prompt or deferred laser) [17]. After two or three cycles of 16-week intervals, monitoring under local policy can be considered according to the level of retinopathy e.g. digital screening or discharge back into diabetic retinopathy screening service or standard general/medical retinal clinics.

If VA and/or CST deteriorate (≥5-letter deterioration in VA and/or ≥10% deterioration in CST from the ‘stable’ values) during the M&E period, treatment with intravitreal aflibercept should be reinitiated. If treatment is reinitiated, injections should continue every 4 weeks until there is no improvement or worsening after two injections (i.e. stability is re-established).

Aflibercept M&E cannot continue indefinitely; however, it is unlikely that a universally accepted set of stopping criteria that is applicable to all patients exists. A number of panel members stated that it would be reasonable to discontinue M&E if the patient had no recurrent oedema for two consecutive intervals of 16 weeks. At this point, the patient should move into a medical retina clinic, with the frequency of follow-up determined by the severity of retinopathy.

While it is important to identify patients who are eligible for an M&E strategy, it is equally important to identify those patients in whom ongoing aflibercept treatment and 4-weekly monitoring is required, and in whom reducing treatment and/or monitoring frequency may compromise outcomes. Indeed, in neovascular AMD, VA gains with ranibizumab seen in PRN trials were not replicated in real-world practice, possibly because of clinics adapting PRN protocols i.e. without 4-weekly monitoring [18].

When following a Protocol T-like aflibercept regimen, monitoring is recommended at a minimum at baseline, 12 weeks, 24 weeks, and whenever a treatment decision is made. More frequent monitoring may be required in patients with severe diabetic retinopathy, bilateral disease or other risk factors.

#### Recommended modifications for Protocol T aflibercept regimen in practice

**Fig. 2:**
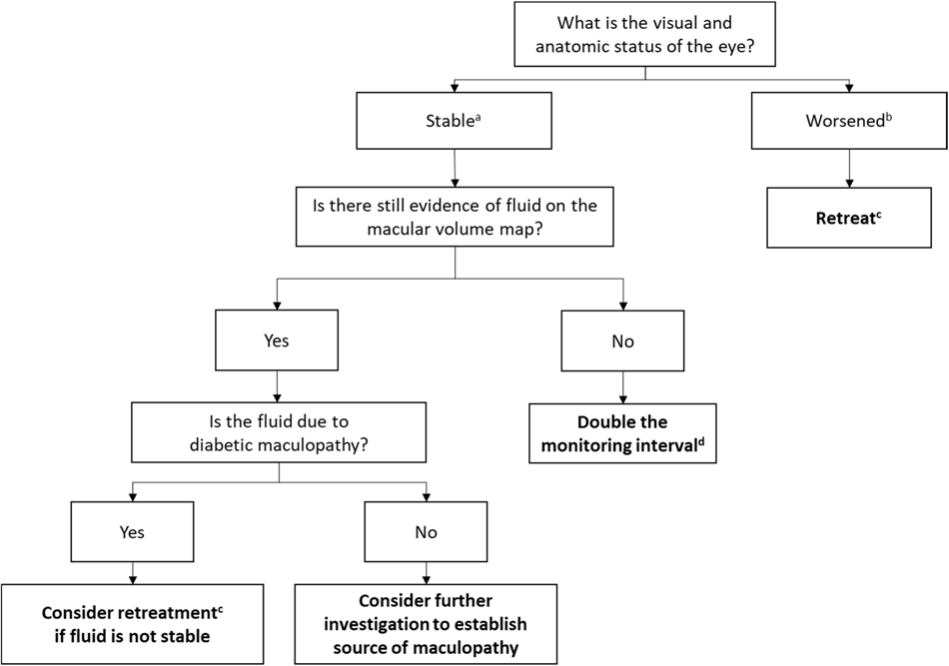
Criteria for decision making during aflibercept monitor-and-extend. ^a^Stability defined as no improvement or worsening in both VA and CST after two consecutive injections. Improvement defined as ≥5-letter improvement in VA and/or ≥10% improvement in CST from values at the start of monitor-and-extend i.e. the point at which the patient was considered stable and the decision was made to defer injections, and worsening defined as ≥5-letter decrease in VA and/or ≥10% increase in CST from values at the start of monitor-and-extend i.e. the point at which the patient was considered stable and the decision was made to defer injections; ^b^Worsening defined as ≥5-letter decrease in VA and/or ≥10% increase in CST from values at the start of monitor-and-extend i.e. the point at which the patient was considered stable and the decision was made to defer injections; ^c^If retreatment is initiated, injections should continue every 4 weeks until there is no improvement or worsening after two injections (i.e. stability is re-established); ^d^Every 4 weeks extended to every 8 weeks then every 16 weeks, if applicable, up to a maximum of 16 weeks. After two or three cycles of 16-week intervals, consider monitoring under local policy e.g. digital screening or discharge back into diabetic retinopathy screening service or standard general/medical retinal clinics. *CST* central subfield thickness, VA visual acuity

The retreatment criteria used up to week 20 in Protocol T are VA of 20/20 and a CST of <250 µm. The panel members suggested a more pragmatic approach for clinical practice, given that patients are unlikely to achieve such targets in the early stages of treatment. The decision on whether to retreat or not during aflibercept M&E should therefore be based on whether VA and CST are stable (with the macular volume map as an additional consideration; level 5 evidence [expert opinion]) or whether they have worsened (Fig. 2).

As outlined above, for patients with DMO treated with aflibercept, changes in the macular volume map should be considered alongside VA and CST. Such changes are qualitative and based on ‘eyeballing’ i.e. looking for evidence of worsening/improvement or recurrence of DMO. As a result, no target values are suggested. Worsening of fluid on the macular volume map may suggest that prolonged treatment or additional treatment is required. Further study is required to identify the threshold for management decisions.

The treating physician should establish that the increase in retinal volume is indeed due to DMO rather than other diabetic retinal pathology. Factors other than DMO–for example, epiretinal membranes (ERM), haemorrhage and cotton wool spots–can also increase macular volume. Fluorescein angiography should therefore be considered prior to administering further anti-VEGF treatment, to guide the treatment decision.

### Sub-optimal response to aflibercept treatment: continue with aflibercept but consider additional treatments

It should be noted that aflibercept is not licensed for use in combination with any other drug treatments or laser [4].

Additional treatments should be considered for patients with a sub-optimal response to aflibercept at the end of the loading phase. The criteria for sub-optimal response are improvement (as for ‘good response’), but with additional concerns that suggest additional treatment may be warranted e.g. worsening of fluid according to the macular volume map (Fig. 1).

There is a broad range of treatments in the DMO armamentarium and these should be utilised. OCT and fundus fluorescein angiography may help to guide the decision of which additional treatment is most appropriate to use.

Managing sub-optimal response to aflibercept is complex and should involve senior medical review.

#### Laser

The panel members agreed that laser may offer additional benefit in patients receiving aflibercept. Targeted focal laser is preferable to grid laser, and the best results may be seen when laser is given after aflibercept i.e. when the macula is drier and the fovea can be visualised more easily. The panel members suggested that the ideal time to administer laser may be around 2 weeks after the aflibercept injection. Fluorescein angiography should be performed before treating with laser to identify potential target areas.

#### Steroids

Dexamethasone and fluocinolone acetonide intravitreal implants may have a role as additional treatments in patients receiving aflibercept. However, in England, Wales and Northern Ireland, dexamethasone is only recommended by NICE in pseudophakic patients in whom a non-corticosteroid treatment has not worked or is unsuitable [8, 19]. Similarly, in Scotland, dexamethasone is accepted for use by the SMC for the treatment of adult patients with visual impairment due to DMO who are pseudophakic or who are considered insufficiently responsive to, or unsuitable for, non-corticosteroid therapy [20].

In England, Wales and Northern Ireland, fluocinolone acetonide is recommended by NICE in pseudophakic patients and only if the manufacturer provides fluocinolone acetonide intravitreal implant with the discount agreed in the patient access scheme [21, 22]. In Scotland, fluocinolone acetonide is accepted for use by the SMC treatment of vision impairment associated with chronic DMO considered insufficiently responsive to available therapies [23].

A recent systematic review concluded that combination treatment with an anti-VEGF plus a steroid does not offer additional benefit compared with monotherapy and is associated with a higher rate of cataract and increased intra-ocular pressure than anti-VEGF monotherapy [24]. There is also a risk of uncontrolled glaucoma when patients fail to attend follow-up appointments. Therefore, intravitreal steroids should only be considered as second-line therapy after aflibercept if the persistent oedema is responsible for the decline in vision and the patient is likely to be compliant with follow-up appointments.

### No response to aflibercept treatment: consider switching from aflibercept to a different treatment

Switching from aflibercept to another treatment should be considered for patients who, following an intensive proactive loading phase with aflibercept, either have no change in VA and/or CST, or meet the ‘worsening’ criteria used in Protocol T (≥5-letter decrease in VA and/or ≥10% increase in CST from baseline; Fig. 1).

The presence of fluid (particularly stable fluid) should not on its own be a reason to switch. In VIVID/VISTA, the ‘saw-tooth’ pattern seen in the aflibercept 2q8 arm for central subfield thickness (CST) was not observed for BCVA [12], suggesting that DMO patients can tolerate some fluid. A sub-analysis from Protocol T also showed that VA gains were achieved whether patients had persistent fluid or not [25]. This enables a little more flexibility in management, in contrast to neovascular AMD patients where the timing of injections needs to be more rigid.

Before considering switching it is important to exclude other causes of poor response (such as vitreomacular traction and/or ERM formation that may potentially benefit from vitrectomy) and differentiate them from true persistent macular oedema. Other potential causes of poor response (e.g. poorly maintained blood pressure or concomitant use of glitazone medications) should be considered. Multimodal imaging should be employed to establish the reason for poor response e.g. fluorescein angiogram to determine any areas of focal leakage that may respond to adjunctive focal laser and to exclude visual loss due to macular ischaemia.

Evidence to support switching from aflibercept to another anti-VEGF is not definitive; therefore, before considering switching to another anti-VEGF, alternative aflibercept regimens and/or additional treatments should be considered.

As for sub-optimal response, non-response to aflibercept is complex and further management should involve senior medical review.

### Other considerations

#### Arterial thromboembolic event (ATE)

The aflibercept SmPC notes that there is a theoretical risk of ATEs, including stroke and myocardial infarction, following intravitreal use of anti-VEGF inhibitors [4]. Several panel members recommended that, if a patient experiences an ATE, aflibercept treatment should be deferred for 12 weeks (based on clinical experience i.e. level 5 evidence), with active monitoring to capture any significant deterioration and to enable treatment to be restarted promptly if sight-threatening changes are noted. However, there was not universal agreement among the panel members.

A patient who has experienced an ATE during aflibercept treatment should be re-counselled by the treating ophthalmologist and should be reminded that anti-VEGFs may potentially increase the incidence of ATE, though the extent of the effect is currently unknown.

#### Bilateral disease

In patients with bilateral disease, the worst eye should drive the treatment or monitoring interval. There are no known safety concerns with injecting both eyes at the same visit. However, to reduce the risk of drug error, it is recommended that both eyes should receive the same anti-VEGF treatment. In the case of compounded products, different anti-VEGF batch numbers should be used for each eye. It should be noted that compounded medicines are not licensed for use in the eye.

## Conclusions

Early aggressive treatment of visual impairment due to DMO in Year 1 is likely to lead to benefits in Year 2 and beyond. Clinical experience supports the evidence base and reinforces that, when starting aflibercept, treatment should begin with intensive proactive dosing. The majority of attendees used six initial 4-weekly doses in line with Protocol T [5], rather than the five initial monthly doses as recommended in the SmPC [4].

At the 24-week visit, response to aflibercept treatment should be assessed in order to determine continued management. For patients with a good response, aflibercept injections should continue every 4 weeks until stability has been established (defined as no improvement or worsening over two consecutive visits, as in Protocol T [5]). Following stability, an aflibercept M&E regimen can be followed, with doubling of the monitoring visits up to a maximum of 16 weeks (note this is not in line with aflibercept SmPC posology, which recommends one injection every 2 months after the loading phase [4]).

Those with a sub-optimal response should continue with aflibercept but additional treatments should be considered (note aflibercept is not licensed for use in combination with any other drug treatments or laser [4]). For patients with no response to aflibercept treatment, switching from aflibercept to a non-anti-VEGF treatment should be considered.

The panel recommends that each institution considers each aflibercept regimen and agrees on which pathway is best to follow, and if there are any exceptions to a particular pathway. For patients who do not fall within these recommendations, DMO management is complex and therefore senior medical input should be sought.

Throughout management, the patient should be kept fully informed of the treatment plan in order to set their expectations and determine treatment priorities and preferences. Tips for effective service delivery during aflibercept treatment are shown in Table 3.

**Table 3:** Tips for effective service delivery during treatment with aflibercept

• Develop separate pathways for anti-VEGF, laser and steroid treatments
• Ideally, manage patients in a dedicated DMO clinic, or alternatively in a dedicated anti-VEGF clinic, with clinicians who have appropriate expertise in managing DMO
• As an institution, consider each aflibercept regimen and agree on which pathway is best to follow, and if there are any exceptions to a particular pathway
• Make use of two-stop services and virtual clinics, where appropriate, to help to overcome capacity issues
◦ In a one-stop service, OCT/VA and injection are performed on the same day
◦ In a two-stop service, OCT/VA and injection performed on different days, and OCT assessments are made remotely in a ‘virtual’ clinic
◦ A one-stop service can work well in clinics where the assessment and injection teams are optimised and where capacity is not a concern
◦ The success of a two-stop approach may depend on region and the distance that the patient has to travel to the clinic. Furthermore, additional appointments, particularly during the loading phase, may not be prudent, given that patients with DMO are more likely than those with neovascular AMD to miss appointments
◦ Upscaling virtual reviews for non-anti-VEGF patients may help to improve capacity for anti-VEGF patients
◦ Clinicians should be flexible and provide different pathways for patients who are at different stages of treatment; a one-stop service may be suitable for those who require frequent injections initially, while a two-stop service and virtual clinics may be preferred for those unlikely to require ongoing injections e.g. patients who have been stable for some time
• Before initiating aflibercept treatment, set treatment expectations for the patient
◦ Together with the patient, decide which regimen is best for them (bearing in mind local agreed pathways)
◦ Remind the patient that intensive dosing in Year 1 is likely to yield benefits in Year 2 and beyond
◦ Show the OCT map (Appendices Figure 2) to patients to help them visualise current fluid status and treatment goals
◦ Adequate patient counselling should help to ensure good attendance
• Schedule a limited number of aflibercept injections in advance
◦ Patients with DMO have a tendency to miss appointments and this can make scheduling difficult
◦ Compared with patients with neovascular AMD, patients with DMO are usually younger, working, and often have many other clinic appointments to attend. Furthermore, their vision does not deteriorate as quickly, meaning that they may feel less urgency to managing their disease
◦ If an appointment is missed, either offer an additional appointment (and reschedule subsequent planned appointments) or continue with the planned scheduled appointments and do not reschedule
• Remind patients to bring their distance glasses to their appointment
• Measure CFT consistently; Appendix Fig. 2 shows where the CFT measurement should be taken from
• Liaise regularly with diabetes physicians in order to ensure optimal glycaemic and blood pressure control

*AMD* age-related macular degeneration, *CFT* central foveal thickness, *DMO* diabetic macular oedema, *OCT* optical coherence tomography, *VA* visual acuity, *VEGF* vascular endothelial growth factor

It should be reiterated that the above recommendations are limited to aflibercept only. Recommendations for other agents approved for DMO were not discussed during the roundtable meeting and are therefore not covered in the paper.

Several opportunities for additional research were identified: whether there is a threshold of retinal thickness beyond which small changes in thickness may not have a large impact (and whether this threshold differs between patients); whether the parameters for response to aflibercept change depending on the period of treatment (loading versus Year 2 or 3, for example); whether laser has a role for treating pockets of fluid; and whether OCT angiogram could, in the future, have a role for visualising the macula before laser.

## Summary

### What was known before

- EURETINA guidelines on management of visual impairment due to DMO note that, for aflibercept, it is currently ‘an open choice’ as to whether loading doses should be followed by fixed bimonthly injections or a PRN regimen with monthly monitoring.
- This paper describes recommendations from a roundtable of retina experts on the use of aflibercept for management of DMO, based on current evidence and practical experience in the UK.

### What this study adds

- When starting aflibercept, clinical experience reinforces that treatment should begin with intensive proactive dosing.
- After intensive proactive dosing with aflibercept:
  - Patients with a good response but who are not yet stable should continue with 4-weekly aflibercept until stability is reached
  - Patients with a good response and stability should initiate aflibercept monitor-and-extend
  - Those with a sub-optimal response should continue with 4-weekly aflibercept but additional treatments should be considered
  - For patients with no response to aflibercept, switching to a non-anti-vascular endothelial growth factor treatment should be considered

## Appendix

Appendix Fig. 1. Licensed posology for aflibercept in diabetic macular oedema [4]

Appendix Fig. 2. Optical coherence tomography scan (Heidelberg Spectralis) showing location of central foveal thickness (CFT) measurement (inner circle showing 245 µm and 286 µm), and increasing volume of the macular volume map outside of the CFT circle
